# Supplementary material for: High-affinity tamoxifen analogues retain extensive positional disorder when bound to calmodulin
Source: Magn Reson (Gott). 2021 Aug 13;2(2):629–42. doi: 10.5194/mr-2-629-2021 (PMC10539762; doi:10.5194/mr-2-629-2021)
Supplement: The supplement related to this article is available online at: https://doi.org/10.5194/mr-2-629-2021-supplement. [file mr-2-629-supplement.pdf]

Supplement of Magn. Reson., 2, 629–642, 2021
https://doi.org/10.5194/mr-2-629-2021-supplement
© Author(s) 2021. CC BY 4.0 License.

Supplement of

High-affinity tamoxifen analogues retain extensive positional disorder when bound to calmodulin

Lilia Milanesi et al.

Correspondence to: Jonathan P. Waltho (j.waltho@sheffield.ac.uk)

The copyright of individual parts of the supplement might differ from the article licence.

Supporting Information

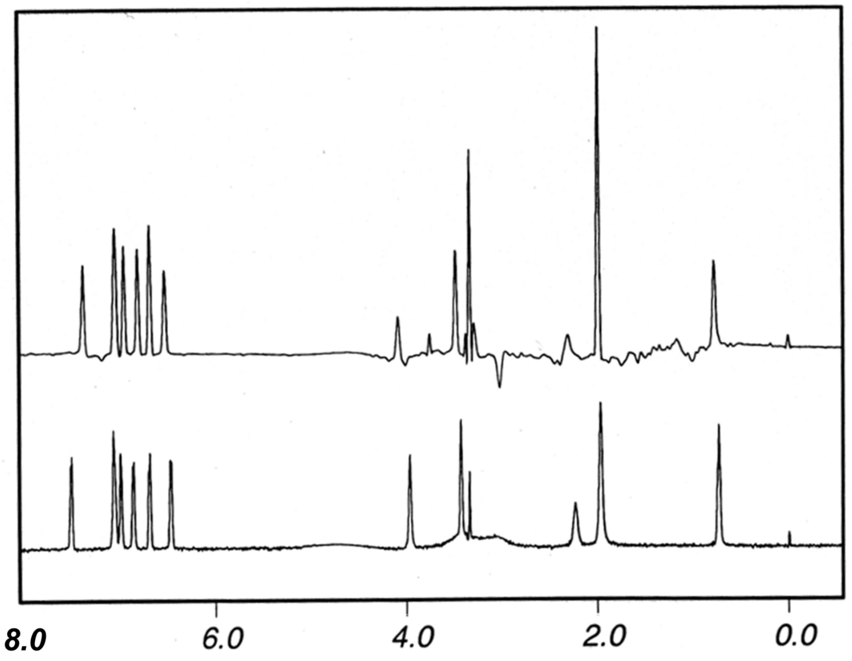

Figure S1: NMR spectra of idoxifene. Lower: ^{1}H spectrum of free idoxifene dissolved in D_2O by addition of 10 M DCl to pH 3.0. An acidic pH is required to generate the protonated form of the ligand as the free base is insoluble in water. The pKa of the pyrrolidine nitrogen of idoxifene is 9.62 (see https://www.ebi.ac.uk/chembldb/compound/inspect/CHEMBL6318) and therefore in the pH range 3.0-6.0 greater than 99% of the ligand is in the protonated form. Upper: a $^{13}C/^{15}N$ filtered spectrum of a 2:1 idoxifene:CaM complex with $^{13}C/^{15}N$ labelled protein (pH 6.0). In the lower spectrum the assignment is (from left to right): H16/18 (7.52 ppm), H8/9/10 (7.06ppm), H7/11 (6.99 ppm), H15/19 (6.86 ppm), H21/25 (6.69 ppm), H22/24 (6.47 ppm), H26 (3.96ppm), H27 (3.43ppm), H28/H 31(3.39/3.06 ppm), H12 (2.23 ppm), H29/30 (1.96), H13 (0.73). The numbering of hydrogens in idoxifene is shown in Figure 1.

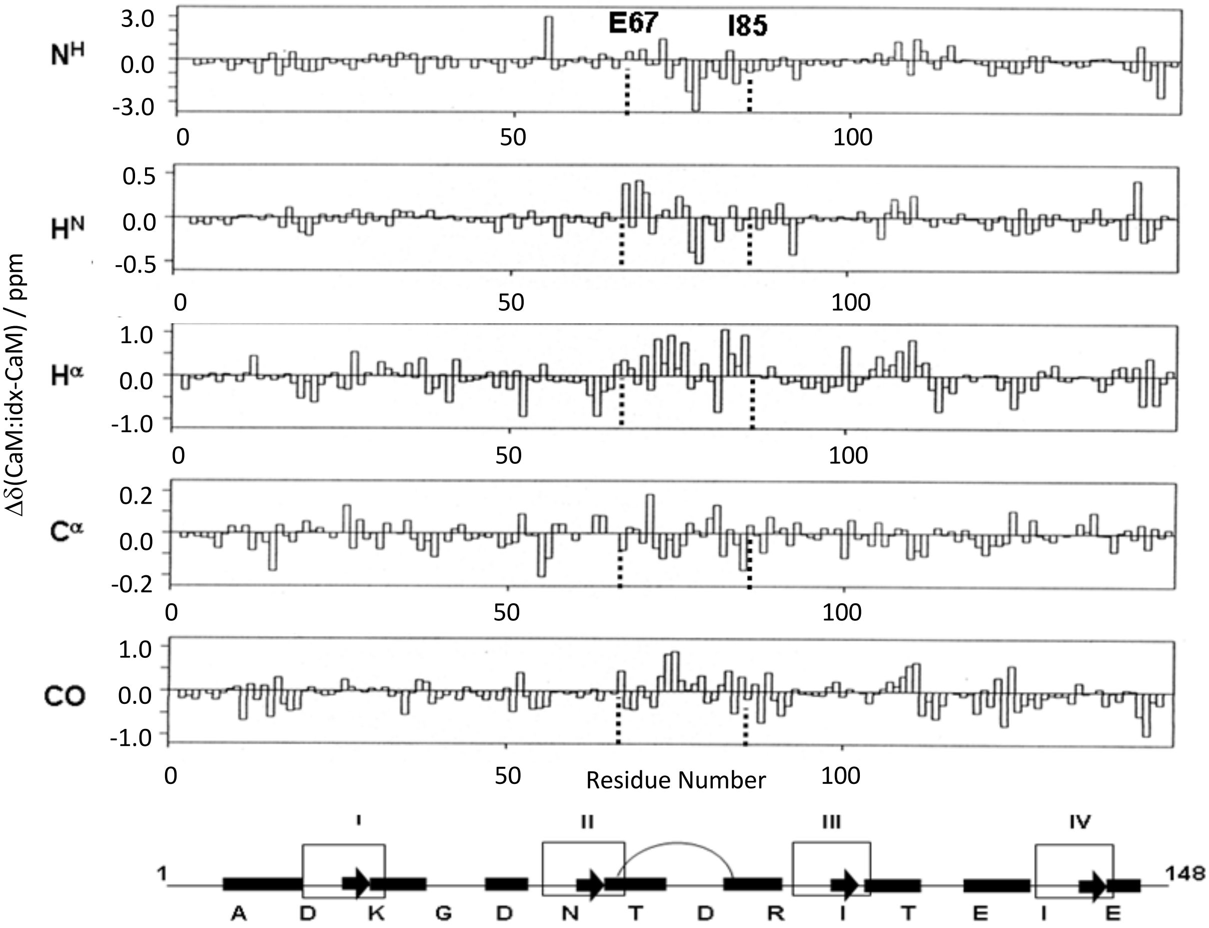

Figure S2: Chemical shifts changes of the five backbone atoms of CaM upon complexation with idoxifene. The graph plots the differences in chemical shift between the complex and the unbound protein as a function of residue number. Dotted lines mark the region E67-I85, which includes residues at the centre of the flexible tether that show remarkable similarity with the chemical shifts changes observed upon complexation of CaM with M13 (see Fig. 2 in Ikura et al., 1991). The amide proton shift differences of residues D78 and D80 are -0.5 and -0.08, respectively. These values, including the direction of shift changes, are very similar to the equivalent shifts observed for the CaM:W7 complex (see Fig. 6 in Osawa et al., 1999). Residues are also identified on the primary sequence of CaM at the bottom of the graphs. Secondary structure elements are indicated above the primary sequence as solid boxes for α-helical regions, solid arrows for β-strands and thin lines for unstructured regions. The curved thin line indicates residues at the centre of the flexible tether (E67-I85) and the rectangular boxes labelled with Roman numerals indicate the four calcium binding loop regions.

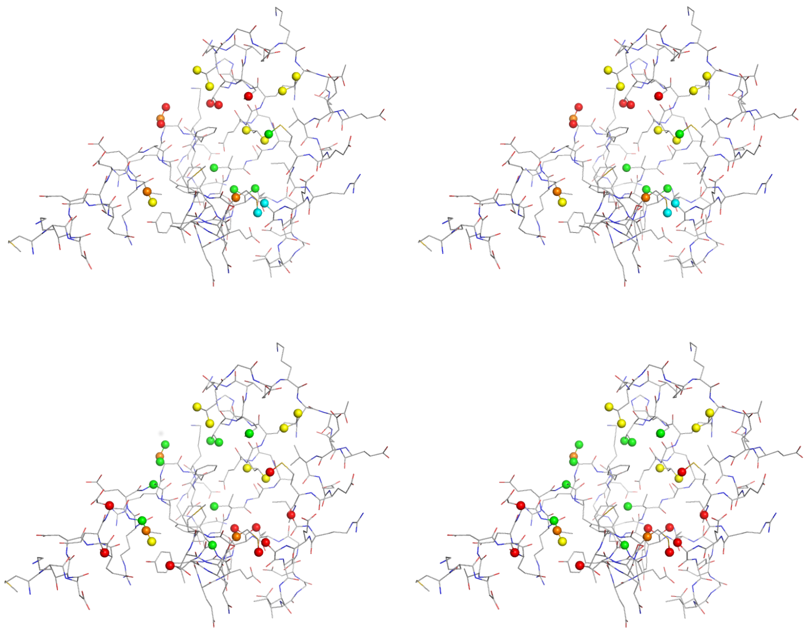

Figure S3: Stereo representation of the heavy atoms of tr2c showing the groups (marked with spheres) that have intermolecular NOEs to (upper) the *p*-iodo-phenyl and (lower) the phenyl group of idoxifene. When one idoxifene molecule is docked into tr2c using NOE restraints, the lowest energy structure has NOE violations to the groups coloured red, satisfies the constraints to the groups coloured green, and satisfies the constraints to at least one nucleus in the groups coloured yellow, cyan and orange. (The idoxifene molecule is not shown for clarity.)

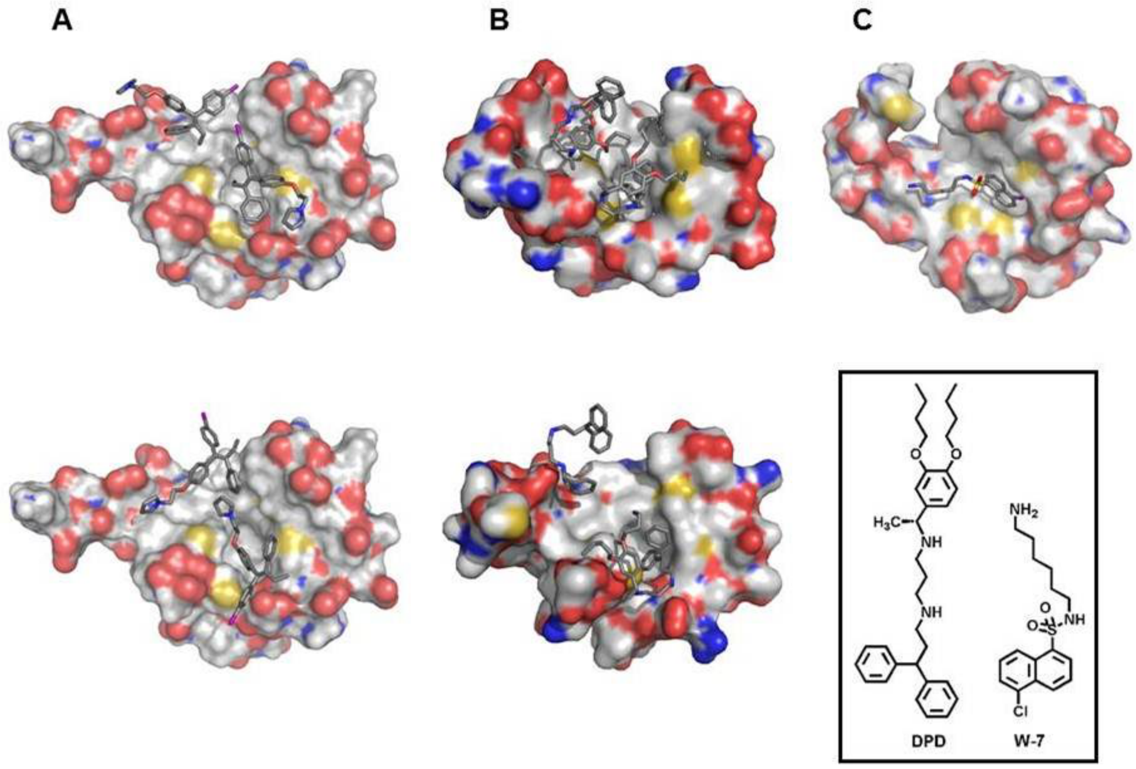

Figure S4: Structures of idoxifene:CaM complexes. *(A)* Representative positions of idoxifene molecules relative to tr2c (pdb entry 1cll). The four orientations shown represent clusters of conformations that satisfy the NOE restraints between idoxifene and the tr2c domain of CaM. The conformations are paired in the diagrams for clarity only. (*B*) The two X-ray structures of CaM:DPD; coordinates taken from pdb entry 1qiv (*top*) and pdb entry 1qiw (*bottom*). In both structures only the tr2c domain is shown. (*C*) The structure of the tr2c:W7 complex obtained from the coordinates of the CaM:W7 complex (pdb entry 1mux). Only the structure of the first deposited model is shown for clarity. Only surface representations are shown to illustrate the variation in degree of occupancy of the hydrophobic pockets and extent of domain closure of the various complexes. The latter can be appreciated by the extent of kink of the N-terminal portion of tr2c (corresponding the left hand side of each diagram). The surface representation is coloured as follows: carbon (*grey*), nitrogen (*blue*), oxygen (*red*) and sulphur (*yellow*). The rectangular box shows the 2D structures of DPD and W-7.

References

Ikura, M., Kay, L. E., Krinks, M., and Bax, A.: Triple-resonance multidimensional NMR-study of calmodulin complexed with the binding domain of skeletal-muscle myosin light-chain kinase - Indication of a conformational change in the central helix, Biochemistry, 30, 5498-5504, doi:10.1021/bi00236a024, 1991.

Osawa, M., Kuwamoto, S., Izumi, Y., Yap, K. L., Ikura, M., Shibanuma, T., Yokokura, H., Hidaka, H., and Matsushima, N.: Evidence for calmodulin inter-domain compaction in solution induced by W-7 binding, FEBS Lett., 442, 173-177, doi:10.1016/s0014-5793(98)01637-8, 1999.
